# tPA-MMP-9 Axis Plays a Pivotal Role in Mobilization of Endothelial Progenitor Cells from Bone Marrow to Circulation and Ischemic Region for Angiogenesis

**DOI:** 10.1155/2016/5417565

**Published:** 2016-08-16

**Authors:** Steve Leu, Yuan-Ji Day, Cheuk-Kwan Sun, Hon-Kan Yip

**Affiliations:** ^1^Institute for Translational Research in Biomedicine, Kaohsiung Chang Gung Memorial Hospital, Kaohsiung 833, Taiwan; ^2^Department of Biotechnology, College of Life Science, Kaohsiung Medical University, Kaohsiung 807, Taiwan; ^3^Department of Anesthesiology, Chang Gung Memorial Hospital and Graduate Institute of Clinical Medical Sciences, Chang Gung University, Taoyuan 333, Taiwan; ^4^Department of Emergency Medicine, E-Da Hospital, I-Shou University, Kaohsiung 824, Taiwan; ^5^Division of Cardiology, Department of Internal Medicine, Kaohsiung Chang Gung Memorial Hospital and Chang Gung University College of Medicine, Kaohsiung 333, Taiwan; ^6^Center for Shockwave Medicine and Tissue Engineering, Kaohsiung Chang Gung Memorial Hospital, Kaohsiung 833, Taiwan; ^7^Department of Medical Research, China Medical University Hospital, China Medical University, Taichung 404, Taiwan; ^8^Department of Nursing, Asia University, Taichung 413, Taiwan

## Abstract

We examined the role of tissue plasminogen activator- (tPA-) matrix metalloproteinase- (MMP-) 9 in mobilizing endothelial progenitor cells (EPCs) from bone marrow to circulation and critical limb ischemia (CLI) region. Male C57BL/6J mice having been irradiated were categorized into wild-type mice (WT) receiving WT bone marrow cell (BMC) transfusion (group 1), WT mice receiving MMP-9 knockout (MMP-9^−/−^) BMC (group 2), MMP-9^−/−^ receiving MMP-9^−/−^ BMC (group 3), and MMP-9^−/−^ receiving WT BMC (group 4), each of which was subdivided into sham control (SC), CLI, SC-tPA, and CLI-tPA. In groups 1 and 4, by post-CLI 18 h and day 14, circulating EPC (C-kit+/CD31+, Sca-1+/KDR+) levels were highest in CLI-tPA subgroup. In groups 2 and 3, EPC levels did not differ among all subgroups. The EPC levels in bone marrow were higher in groups 2 and 3 than those in groups 1 and 4. By day 14, in animals with CLI, expression levels of proangiogenic factors (CXCR4, SDF-1*α*, and VEGF) showed similar trends as circulating EPC levels. Moreover, the number of infiltrated neutrophils and macrophages in quadriceps was higher in groups 1 and 4 than groups in 2 and 3. In conclusion, tPA-MMP-9 axis plays a crucial role in EPC mobilization and angiogenesis in experimental CLI.

## 1. Introduction

Endothelial progenitor cells (EPCs) are originally identified as bone marrow- (BM-) derived endothelial precursor cells that contribute to neovascularization [1]. In normal condition, most EPCs locate within the stem cell niche in bone marrow and only few circulating populations in the peripheral blood [1, 2]. To enhance the mobilization of bone marrow-derived EPCs, it is considered as an effective strategy to enhance EPC-mediated injury recovery postischemic damage [3, 4]. Studies have previously demonstrated mobilization of endothelial progenitor cells (EPCs) from bone marrow (BM) to circulation and their homing to ischemic region to enhance angiogenesis/vasculogenesis through upregulation of metalloproteinase- (MMP-) 9 [5] activity and suppression of the CD26/dipeptidyl peptidase IV (DPP IV) system [6], thereby improving ischemia-related organ dysfunction [5, 6]. Recently, we have demonstrated that tissue plasminogen activator (tPA) could augment the number of circulating EPCs, angiogenesis, and blood flow to ischemic tissue in a murine model of critical limb ischemia (CLI) [7]. Further analysis in our study has revealed that an increase in circuiting EPC and enhancement of angiogenesis in ischemic zone are mainly attributable to an elevation of circulating and a reduction of bone marrow (BM) SDF-1*α* concentration, accompanied by upregulation of MMP-9 activity in BM [7]. By using a tPA knockout mouse model, we have further identified the essential role of endogenous tPA in augmenting circulating EPCs, angiogenesis, and blood flow through regulating MMP-9 activity in the ischemic limb in a murine model [8]. However, although these studies [5–8] consistently emphasized the relationship between MMP-9 activity and the mobilization of EPCs from BM to circulation, the precise mechanisms involved in the regulation of the kinetics of EPC mobilization have not been thoroughly clarified in these studies [5–8].

Fascinatingly, some previous studies have identified that MMP-9 acts by cleaving the membrane-bound c-Kit ligand (c-Kit-L) to the soluble form c-Kit-L, which then interacts with the EPCs c-Kit receptor to initiate the signal (i.e., the downstream signaling of MMP-9) that is crucial for BM-EPC differentiation and mobilization to systemic circulation [9–12]. In addition to EPC, the role of MMP-9 in regulating migration of neuronal and mesenchymal stem cells has also been indicated through cultured cell models and gene deficient mouse model [13, 14]. A recent study also indicated that the levels and activity of MMP-9 were altered in patients with CLI [15]. To further understand whether MMP-9 plays a unique role in tPA-mediated regulation of the mobilization of BM-EPCs into circulation, the MMP-9 deficient mice (MMP-9^−/−^), wild-type (WT) C57BL/6J mice, and BM reconstructions of MMP-9^−/−^ were adopted in an experimental setting of critical limb ischemia (CLI) in the present study to investigate the following issues: (1) to determine the circulating and BM levels of EPCs (c-Kit+/CD31+, Sca-1+/KDR+) in a setting of CLI with and without tPA treatment in both wild-type and MMP-9^−/−^ mice; (2) to compare the circulating and BM levels of EPCs in BM reconstruction of wild-type mice using MMP-9^−/−^ as BM donor and vice versa; (3) to assess the blood flow and angiogenesis in ischemic area of the animals at day 14 after CLI induction; (4) to compare the angiogenesis capacity among WT and MMP-9^−/−^ mice.

## 2. Materials and Methods

### 2.1. Ethics

All animal experimental procedures were approved by the Institute of Animal Care and Use Committee at our institute and performed in accordance with the Guide for the Care and Use of Laboratory Animals (NIH publication number 85-23, National Academy Press, Washington, DC, USA, revised 1996).

### 2.2. Animals, Irradiation, and Bone Marrow Cell (BMC) Transplantation

MMP-9 deficient mice in C57BL/6J background were purchased from The Jackson Laboratory (*Mmp9* ^*tm1Tvu*^, stock number 007084) and bred in the specific pathogen-free (SPF) condition in the Association for Assessment and Accreditation of Laboratory Animal Care- (AAALAC-)certificated experimental animal center within Kaohsiung Chang Gung Memorial Hospital. The procedure and protocol of irradiation and BM transplantation were based on previous descriptions [16]. In detail, 6-week-old male WT C57BL/6J mice (Charles River Technology, BioLASCO Taiwan Co., Ltd., Taiwan) having been irradiated (600 cGy × 2 times; the interval of two irradiation times was 4 hrs) and given immediate BMC transfusion (1.0 × 10^6^) after the 2nd irradiation were divided into group 1 (BMC from WT to WT) and group 2 (BMC from MMP-9^−/−^ mice to WT mice). Animals in each group were then subdivided into four subgroups: (1) sham-operated control (SC receiving only skin incision over left hindlimb), (2) CLI only, (3) SC + tPA (intravenous 4 mg/kg), and (4) CLI + tPA. Six animals were utilized in each subgroup. For comparison, age-matched male MMP-9^−/−^ mice having received the same dosage of irradiation and immediate BMC transfusion (1.0 × 10^6^) were divided into group 3 (BMC from MMP-9^−/−^ to MMP-9^−/−^) and group 4 (BMC from WT to MMP-9^−/−^). Similarly, animals in groups 3 and 4 were also categorized into four subgroups (*n* = 6 for each subgroup). For determining the BM levels of EPCs, 4 additional animals in each subgroup were utilized and were euthanized at 18 h after CLI induction. The experimental grouping and procedure are presented in Supplemental Figure 1 in Supplementary Material available online at http://dx.doi.org/10.1155/2016/5417565.

### 2.3. Animal Model of Critical Limb Ischemia

By the end of two months (i.e., 60 days) after irradiation, both MMP-9^−/−^ and WT mice, weighing 25–30 gm, were anesthetized with inhalational 2.0% isoflurane. The mice receiving CLI only and CLI + tPA were placed in a supine position on a warming pad at 37°C with the left hindlimbs shaved. Only sham operation was done for SC and SC + tPA animals. Under sterile conditions, the left femoral artery, small arterioles, and circumferential femoral artery were exposed and ligated over their proximal and distal portions before being excised. To get rid of all collateral circulation, all branches were also removed. However, the veins were left intact during the procedure. After the procedure, the wound was closed and the animal was allowed to recover from anesthesia in a portable animal intensive care unit (ThermoCare®) for 24 hours.

### 2.4. The Rationale of tPA Dosage in the Study

A single dose of intravenous tPA was given at 3 h after CLI induction. The dosage of tPA in the present study was according to our recent reports [7, 8]. The rationale of adopting a tPA dosage of 4.0 mg/kg has been described in detail in our previous study [7].

### 2.5. Measurement of Blood Flow with Laser Doppler in both Normal and CLI Regions

The procedure and protocol of examination of blood flow using Laser Doppler was based on our recent studies [7, 8]. Briefly, assessment of blood flow over the normal and ischemic limbs for each animal was performed under anesthesia with inhalational 2.0% isoflurane prior to the CLI procedure and on days 1 and 14 after CLI induction prior to being euthanized. Each animal was placed in a supine position on a warming pad at 37°C with both hindlimbs thoroughly shaved. The blood flow was then assessed using a Laser Doppler scanner (moorLDLS, Moor, Co. UK). All data were collected and put into a computer for further analysis. Following blood flow measurement on day 14 after CLI induction, the mice were euthanized and the quadriceps muscle was collected for individual study.

### 2.6. Flow Cytometric Quantification of Circulating and Bone Marrow Endothelial Progenitor Cells

The procedure and protocol of flow cytometric analysis was according to our recent reports [7, 8]. In detail, peripheral blood sampling at baseline and at 18 h and on day 14 after CLI induction was obtained via cardiac puncture with a 30# needle. Moreover, the BM levels of EPCs were measured at 18 h and on day 14 after CLI induction by needle aspiration and washing from the long bone of the animals. After treatment with red blood cell-lysing buffer, the cells were labeled with appropriate antibodies. Flow cytometric quantification of EPCs through identification of the chosen cell surface markers was performed based on our recent reports [7, 8]. Briefly, the cells were incubated for 30 minutes with primary antibodies, including PE-conjugated antibodies (against Sca-1 and CD31, BD Biosciences), FITC-conjugated antibody against c-Kit (BD Biosciences), and anti-KDR (NeoMarkers) antibodies which were further recognized by Alexa flour 488-conjugated secondary antibodies (Invitrogen). Isotype-identical antibodies (IgG) served as controls. Flow cytometric analyses were performed by utilizing a fluorescence-activated cell sorter (Beckman Coulter FC500 flow cytometer).

### 2.7. Western Blot for Protein Expression of Angiogenesis Factors

The procedure and protocol of western blot were according to our recent reports [7, 8]. Briefly, equal amounts (10–30 *μ*g) of protein extracts from ischemic quadriceps of the animals (*n* = 6 for each group) were loaded and separated by SDS-PAGE using 7% or 12% acrylamide gradients. The membranes were incubated with monoclonal antibodies against CD31 (1 : 1000, Abcam), CXCR4 (1 : 1000, Abcam), vascular endothelial growth factor (VEGF) (1 : 1000, Abcam), and stromal cell-derived growth factor- (SDF-) 1*α* (1 : 1000, Cell Signaling). Signals were detected with HRP conjugated goat anti-rabbit IgG. Proteins were transferred to nitrocellulose membranes which were then incubated in the primary antibody solution (anti-DNP 1 : 150) for two hours, followed by incubation with secondary antibody solution (1 : 300) for one hour at room temperature. The washing procedure was repeated eight times within 40 minutes. Immunoreactive bands were visualized by enhanced chemiluminescence (ECL; Amersham Biosciences) which was then exposed to Biomax L film (Fuji). For quantification, ECL signals were digitized using Labwork software (UVP).

### 2.8. Immunofluorescent (IF) and Immunohistochemical (IHC) Staining

For IF staining, cryosections (10 *μ*m) of quadriceps were fixed and permeated with acetone or 4% paraformaldehyde with 0.5% Triton X-100. IF staining was performed for the examinations of CD31+, CXCR4+, SDF-1*α*+, CD11+ (neutrophil marker), and CD68+ (macrophage marker) cells using respective primary antibodies based on our recent studies [7, 8]. Irrelevant antibodies were used as controls in the current study. For IHC staining, fixed and permeated sections were incubated with primary antibody against alpha-smooth muscle actin (*α*-SMA) (1 : 400, Millipore) at room temperature for 1 hour, followed by incubating with anti-mouse-HRP conjugated secondary antibody for 30 minutes at room temperature. Signals were encolored with 3,3′ diaminobenzidine (DAB) (0.7 gm/tablet) (Sigma). Finally, hematoxylin was added as a counterstain for nuclei. For quantification, three quadriceps sections were analyzed in each mouse. For quantification, three randomly selected HPFs (high power fields) were analyzed in each section. The mean number per HPF for each animal was then determined by summation of all numbers divided by 9.

### 2.9. Determination of the Impact of MMP-9 on* Ex Vivo* Angiogenesis

To assess the impact of MMP-9 on angiogenesis* ex vivo*, both WT and MMP-9^−/−^ mouse aortic rings (from mouse ascending aorta) were cultured in M199 culture medium for 5 days, followed by determination of the number of sprouts from each aortic ring.

### 2.10. Method for Determining Mouse Aortic Ring Angiogenesis

Aortic ring angiogenesis was conducted in fifteen-well tissue culture slide which contained 20 *µ*L of 10 mg/mL matrigel (BD Biosciences, NJ) 30 *µ*L endothelial cell medium (ECM) (Sciencell). The plates were allowed to gel for 40 minutes at 37°C and 5% CO_2_. Thoracic aortas were excised from 10-week-old WT and MMP-9^−/−^ mice, followed by removal of all extraneous tissue and branching vessels with forceps and a scalpel. The aorta was cut into cross sections at 1 mm intervals and embedded in matrigel-coated wells filled with 30 *µ*L ECM medium. Two aortic rings of each mouse were utilized for angiogenesis assay. These rings were incubated for 5 days at 37°C and 5% CO_2_ and photographed at day 5 with 40.0x magnification. The number and length of sprouting vessels were quantified by Wimsprout (Wimasis) software.

### 2.11. Zymography Analysis

For zymography, supernatants from bone marrow were collected and centrifuged (500 ×g, 5 min) to remove cells and debris. Protein extract was electrophoresed in 8% SDS-PAGE containing 0.1% gelatin. After migration and washing, gels were incubated (16 h, 37°C) in activation buffer (50 mM Tris-base at pH 7.5, 5 mM CaCl_2_, 0.02% NaN_3_, and 1 *μ*M ZnCl_2_). Gels were stained with Coomassie staining solution (0.5% Coomassie, 50% MeOH, 10% acetic acid, and 40% H_2_O) for 90 minutes, followed by destaining (0.5% Coomassie, 50% MeOH, 10% acetic acid, and 40% H_2_O).

### 2.12. Statistical Analysis

Data was expressed as mean values (mean ± SD). The significance of differences between two groups was evaluated with *t*-test. The significance of differences among groups was evaluated using one-way ANOVA, followed by Bonferroni multiple comparison* post hoc* test. Statistical analysis was performed using Prism 5 statistical software (GraphPad Software, La Jolla, CA, USA). A probability value of less than 0.05 was considered statistically significant.

## 3. Results

### 3.1. Comparison of Angiogenesis Capacity between Wild-Type Mice and MMP-9^−/−^ Mice

**Figure 1:**
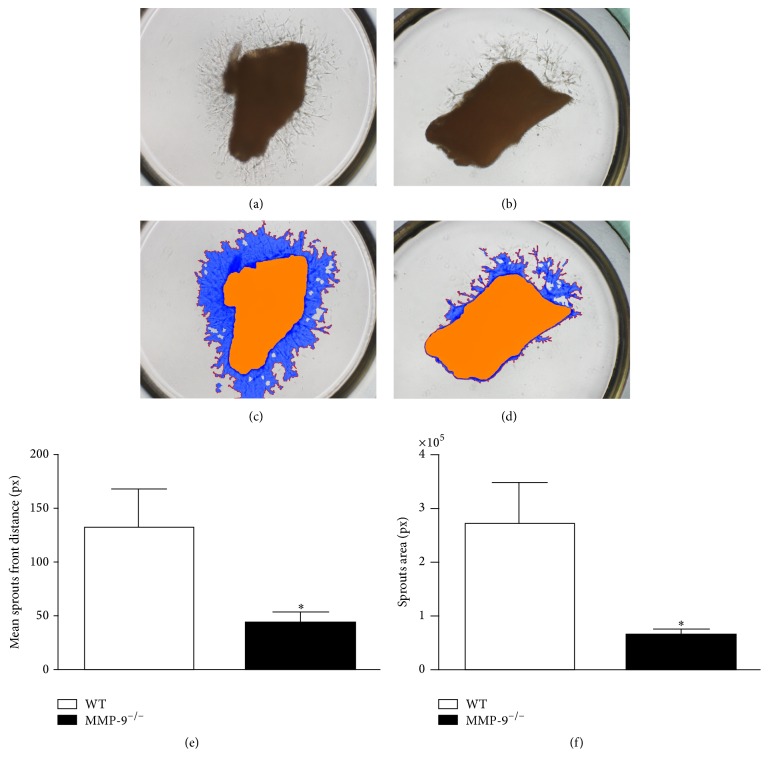
*In vitro* studies for quantification of angiogenesis ability in (a–d) illustrating the results of aortic ring culture in wild-type (WT) (a, c) and MMP-9^−/−^ (b, d) mice, respectively. (e) Analytical result of mean distance of sprouts. (f) Analytical result of mean sprout area. *∗* indicates *p* < 0.0001 between MMP9^−/−^ group and WT group. *n* = 6 for each group.

 In the physiological condition, not only endothelial progenitor cell itself but also surrounding growth factors, inflammatory cytokines, chemokines, and other components of extracellular matrix all contribute in regulating angiogenesis [17]. Hence, instead of tube formation assay of isolated EPCs, we applied aortic ring angiogenesis assays to determine the effects of MMP-9 on angiogenesis. The angiogenesis on aortic ring (i.e., the numbers of sprouts) was significantly reduced in MMP-9^−/−^ mice compared with that in WT mice (Figure 1). This finding suggests that MMP-9 activity is needed for angiogenesis.

### 3.2. Flow Cytometric Quantification of Serial Changes in Circulating Level of c-Kit+/CD31+ and Sca-1+/KDR+ Cells: Subgroup Analysis

**Figure 2:**
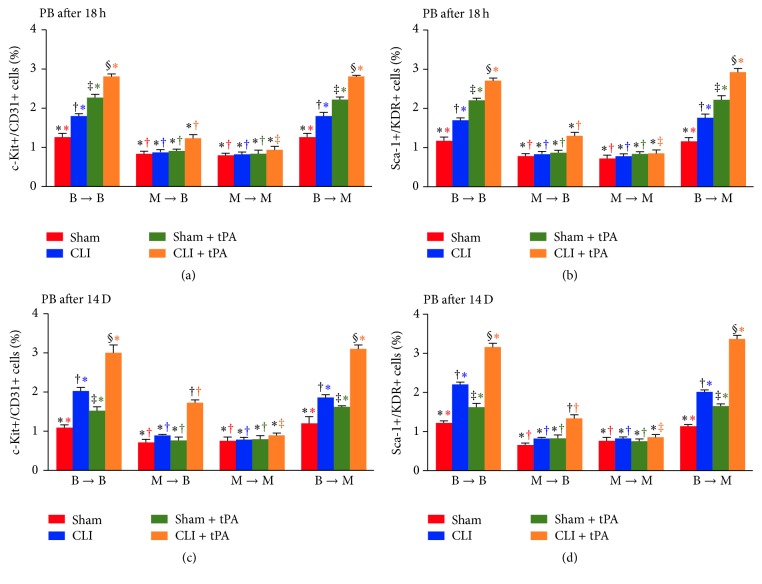
Flow cytometric analysis of circulating level of endothelial progenitor cells (EPCs) at 18 h and on day 14 after critical limb ischemia (CLI) procedure. (Upper panel) Circulating numbers of c-Kit+/CD31+ cells (a) and Sca-1+/KDR+ (b) at 18 h after CLI procedure. *n* = 4 for each group. (Lower panel) Circulating numbers of c-Kit+/CD31+ cells (c) and Sca-1+/KDR+ cells (d) at day 14 after CLI procedure. *n* = 6 for each group. All statistical analyses were performed by one-way ANOVA followed by Bonferroni multiple comparison* post hoc* test. tPA = tissue plasminogen activator. (1) Statistical analysis for subgroups [i.e., sham control (SC), CLI, SC + tPA, and CLI + tPA] of each group [group 1 (WT to WT, i.e., B → B), group 2 (MMP-9^−/−^ to WT, i.e., M → B), group 3 (MMP-9^−/−^ to MMP-9^−/−^, i.e., M → M), group 4 (WT to MMP-9^−/−^, i.e., B → M)]. For subgroups with different black symbols (*∗*, †, ‡, and §), *p* < 0.05. (2) Statistical analysis for group comparison with respective treatment condition (comparing among bar charts with the same color). (1) For SC subgroup (red bar chart), groups with different red symbols (*∗*, †), *p* < 0.05. (2) For CLI subgroup (blue bar chart), groups with different blue symbols (*∗*, †), *p* < 0.05. (3) For SC + tPA subgroup (green bar chart), groups with different green symbols (*∗*, †), *p* < 0.05. (4) For CLI + tPA subgroups (orange bar chart), groups with different orange symbols (*∗*, †, and ‡), *p* < 0.05.

In SC mice prior to CLI induction (i.e., baseline condition), the circulating numbers of c-Kit+/CD31+ and Sca-1+/KDR+ cells were significantly higher in groups 1 (WT to WT) and 4 (WT to MMP-9^−/−^) than in groups 2 (MMP-9^−/−^ to WT) and 3 (MMP-9^−/−^ to MMP-9^−/−^), but there was no notable difference between groups 1 and 4 or between groups 2 and 3 (Figure 2).

In CLI, SC + tPA, and CLI + tPA subgroups after CLI induction (i.e., at 18 h and on day 14 after CLI procedure), the circulating numbers of c-Kit+/CD31+ and Sca-1+/KDR+ cells were significantly higher in groups 1 and 4 than in groups 2 and 3, but there was no significant difference between groups 1 and 4. In addition, these parameters also showed no difference between groups 2 and 3 in their SC, SC + tPA, and CLI subgroups. However, these parameters were significantly higher in group 2 than in group 3 when their CLI + tPA subgroups were compared (Figure 2).

### 3.3. Flow Cytometric Quantification of Serial Changes in Circulating Levels of c-Kit+/CD31+ and Sca-1+/KDR+ Cells: Group Analysis

By 18 h after CLI induction, the circulating numbers of c-Kit+/CD31+ and Sca-1+/KDR+ cells in group 1 were highest in the CLI + tPA subgroup and lowest in SC and significantly higher in the SC + tPA than in the CLI subgroups. Besides, these parameters in group 4 showed an identical pattern compared to that of group 1 among the four subgroups (Figures 2(a) and 2(b)). By day 14 after CLI induction, the circulating levels of these biomarkers exhibited a pattern similar to that at 18 h among the animals of groups 1 and 4 except for a significantly reversed change in the expression levels of these parameters between the CLI and SC + tPA subgroups (Figures 2(c) and 2(d)).

By 18 h and day 14 after CLI induction, the circulating numbers of c-Kit+/CD31+ and Sca-1+/KDR+ cells in group 3 did not differ among the SC, CLI, SC + tPA, and CLI + tPA subgroups. However, these parameters in group 2 were significantly higher in the CLI + tPA subgroup than those in the SC, CLI, and SC + tPA subgroups, but there was no difference among the latter three subgroups in group 2 (Figure 2).

### 3.4. Flow Cytometric Subgroup Analysis of Bone Marrow EPC Level at 18 h and on Day 14 after CLI Induction

**Figure 3:**
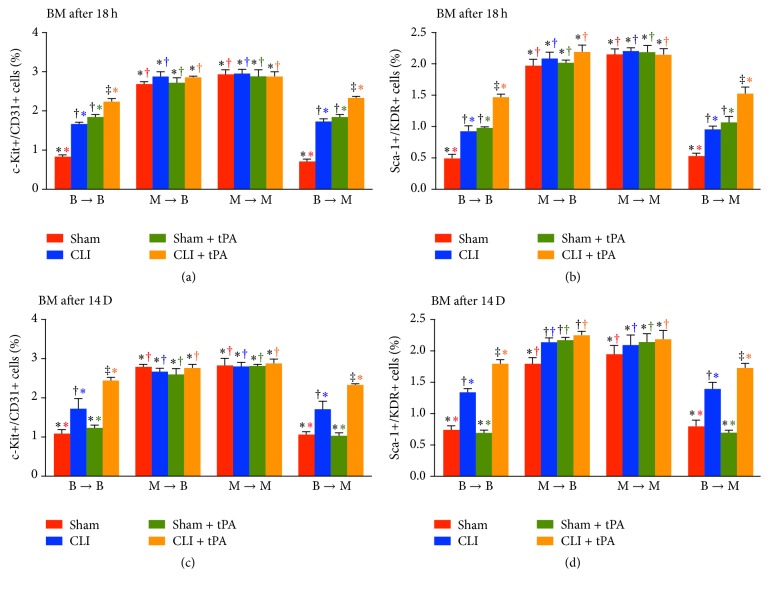
Flow cytometric analysis of bone marrow (BM) level of endothelial progenitor cells (EPCs) at 18 h and on day 14 after critical limb ischemia (CLI) procedure. (Upper panel) Bone marrow levels of c-Kit+/CD31+ cells (a) and Sca-1+/KDR+ (b) at 18 h after CLI procedure. *n* = 4 for each group. (Lower panel) Bone marrow levels of c-Kit+/CD31+ cells (c) and Sca-1+/KDR+ cells (d) at day 14 after CLI procedure. *n* = 6 for each group. All statistical analyses were performed by one-way ANOVA followed by Bonferroni multiple comparison* post hoc* test. tPA = tissue plasminogen activator. (1) Statistical analysis for subgroups [i.e., sham control (SC), CLI, SC + tPA, and CLI + tPA] of each group [group 1 (WT to WT, i.e., B → B), group 2 (MMP-9^−/−^ to WT, i.e., M → B), group 3 (MMP-9^−/−^ to MMP-9^−/−^, i.e., M → M), group 4 (WT to MMP-9^−/−^, i.e., B → M)]. For subgroups with different black symbols (*∗*, †, ‡, and §), *p* < 0.05. (2) Statistical analysis for group comparison with respective treatment condition (comparing among bar charts with the same color). (1) For SC subgroup (red bar chart), groups with different red symbols (*∗*, †), *p* < 0.05. (2) For CLI subgroup (blue bar chart), groups with different blue symbols (*∗*, †), *p* < 0.05. (3) For SC + tPA subgroup (green bar chart), groups with different green symbols (*∗*, †), *p* < 0.05. (4) For CLI + tPA subgroups (orange bar chart), groups with different orange symbols (*∗*, †), *p* < 0.05.

By 18 h and at day 14 after CLI induction, comparison among different groups and their subgroups demonstrated that although the pattern of subgroup changes in BM levels of EPC (i.e., c-Kit+/CD31+ and Sca-1+/KDR+ cells) was similar to that in the circulation for each group, the pattern of changes in EPC prevalence between the BM and circulation among the four groups was reversed (Figures 2 and 3). The prevalence of EPC was significantly higher in the BM environment than that in the circulation in groups 2 and 3, while groups 1 and 4 displayed a reversed pattern (Figures 2 and 3). These findings imply that EPCs were trapped and retained in BM resulting from loss of MMP-9 activity in BM.

### 3.5. Laser Doppler Analysis of Serial Changes of Blood Flow after CLI Induction

**Figure 4:**
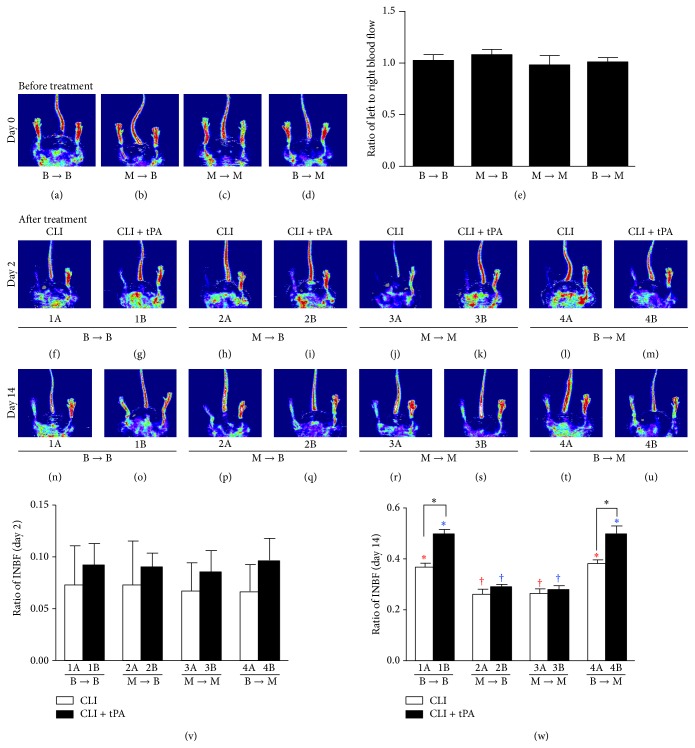
Serial changes of Laser Doppler scanning of hind limb blood flow prior to and after critical limb ischemia (CLI) induction. (a–d) The normal blood flow in both hind limbs in each group prior to the CLI procedure. (e) Ratio of left (ischemia) to right (normal) blood flow, *p* = 1.0. (f–u) The blood flow in left and right hind limbs under the treatment conditions of CLI and CLI + tPA in 4 groups (i.e., B → B, M → B, M → M, and B → M) at day 2 or day 14 after the procedure. (v) Quantitation and calculation of ratio of ischemia to normal blood flow (INBF) among the four groups at day 2. (w) Quantitation and calculation of ratio of INBF among the four groups at day 14. Statistical analysis for individual group at different treatment conditions. (1) In B → B, 1A versus 1B, *∗* indicates *p* < 0.01. (2) In M → B, 2A versus 2B, *p* > 0.5. (3) In M → M, 3A versus 3B, *p* > 0.5. (4) In B → M, 4A versus 4B, *∗* indicates *p* < 0.01. (2) Statistical analysis for group comparison with respective treatment condition. (1) For CLI subgroup (white bar chart), groups with different red symbols (*∗*, †), *p* < 0.01. (2) For CLI + tPA subgroup (black bar chart), groups with different blue symbols (*∗*, †), *p* < 0.01. SC = sham control; tPA = tissue plasminogen activator. *n* = 6 for each group.

Laser Doppler scanning demonstrated no difference in the ratio of ischemic/normal blood flow (INBF) among the four groups of animals prior to (i.e., day 0) CLI induction (Figure 4). By day 2 after CLI induction, although the INBF ratio was significantly reduced in all four groups of animals compared to their baselines, there was no significant difference among the four groups at this time point (Figure 4). By day 14 after CLI, however, the ratio was significantly higher in groups 1 and 4 than that in groups 2 and 3 in their CLI and CLI + tPA subgroups (Figure 4(w)).

By day 14 after CLI induction, in groups 1 and 4, the INBF ratio was significantly higher in the CLI + tPA subgroups than that in the CLI subgroups. However, the ratio did not differ between groups 1 and 4 when their respective subgroups were compared (Figure 4(w)).

### 3.6. Immunofluorescent Staining for Identification of Angiogenesis Cells in Ischemic Quadriceps on Day 14 after CLI Induction: Group Analysis

**Figure 5:**
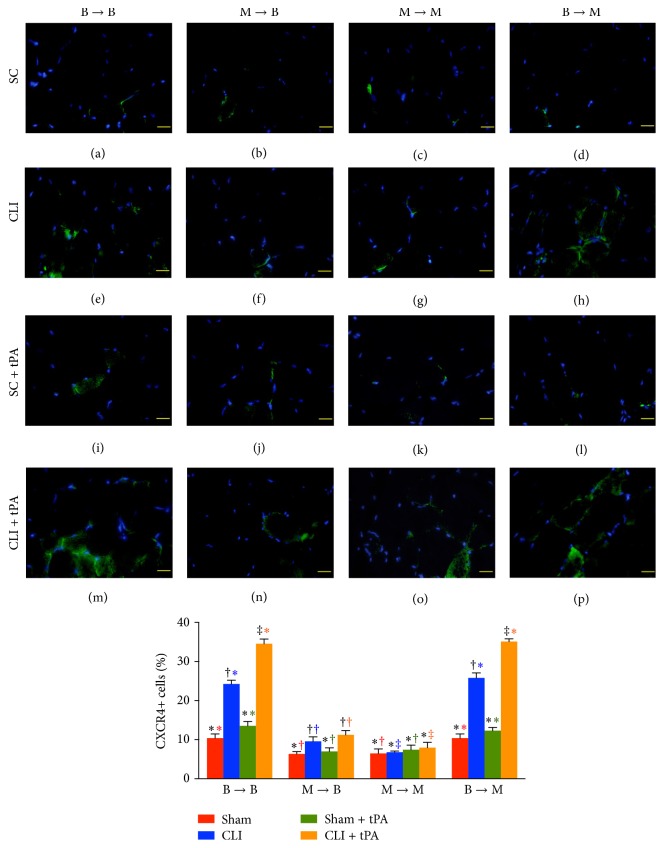
Immunofluorescent (IF) microscopic findings (400x) of CXCR+ cells in ischemic quadriceps at day 14 after critical limb ischemia (CLI) induction. (a–p) IF microscopic findings of CXCR4+ cells in ischemic area in four treatment conditions of four groups. (1) Statistical analysis for subgroups of each group. (1) In B → B group, subgroups with different black symbols (*∗*, †, and ‡), *p* < 0.05. (2) In M → B group, subgroups with different black symbols (*∗*, †, and ‡), *p* < 0.05. (3) In M → M group, *p* > 0.5. (4) In B → M group, subgroups with different black symbols (*∗*, †, and ‡), *p* < 0.5. (2) Statistical analysis for group comparison with respective treatment condition. (1) For SC subgroup (red bar chart), groups with different red symbols (*∗*, †), *p* < 0.05. (2) For CLI subgroup (blue bar chart), groups with different blue symbols (*∗*, †, and ‡), *p* < 0.05. (3) For SC + tPA subgroup (green bar chart), groups with different green symbols (*∗*, †), *p* < 0.05. (4) For CLI + tPA subgroup (orange bar chart), groups with different orange symbols (*∗*, †, and ‡), *p* < 0.05. All statistical analyses were performed by one-way ANOVA followed by Bonferroni multiple comparison* post hoc* test. SC = sham control; tPA = tissue plasminogen activator. *n* = 6 for each group.

**Figure 6:**
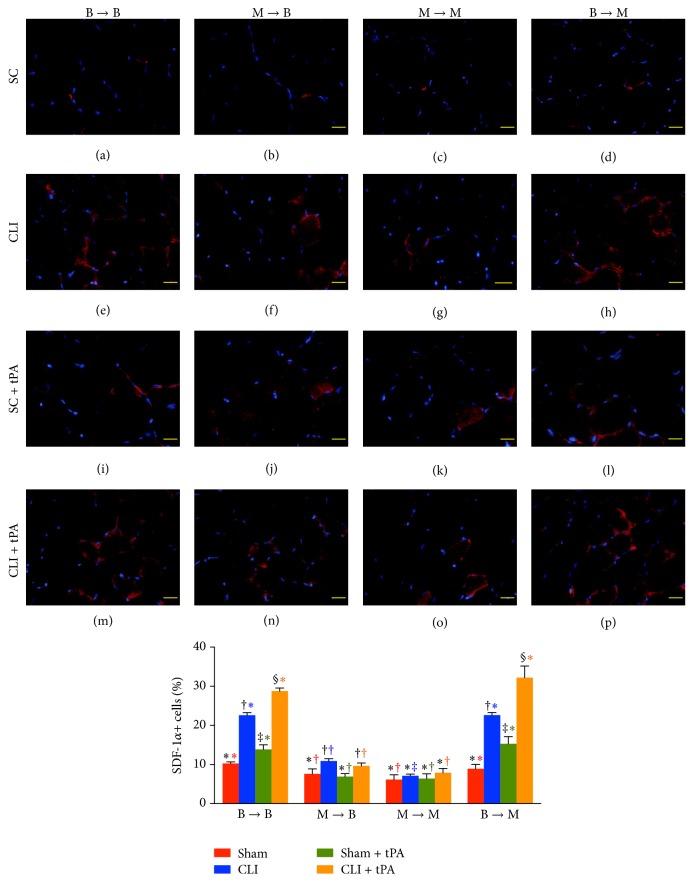
Immunofluorescent (IF) microscopic findings (400x) of stromal cell-derived factor- (SDF-) 1*α*+ cells in ischemic quadriceps at day 14 after critical limb ischemia (CLI) induction. (a–p) IF microscopic findings of SDF-1*α*+ cells in ischemic area in four treatment conditions of four groups. (1) Statistical analysis for subgroups of each group. (1) In B → B group, subgroups with different black symbols (*∗*, †, and ‡), *p* < 0.05. (2) In M → B group, subgroups with different black symbols (*∗*, †), *p* < 0.05. (3) In M → M group, *p* > 0.5. (4) In B → M group, subgroups with different black symbols (*∗*, †, and ‡), *p* < 0.05. (2) Statistical analysis for group comparison with respective treatment condition. (1) For SC subgroup (red bar chart), groups with different red symbols (*∗*, †), *p* < 0.05. (2) For CLI subgroup (blue bar chart), groups with different blue symbols (*∗*, †, and ‡), *p* < 0.05. (3) For SC + tPA subgroup (green bar chart), groups with different green symbols (*∗*, †), *p* < 0.05. (4) For CLI + tPA subgroup (orange bar chart), groups with different orange symbols (*∗*, †), *p* < 0.05. All statistical analyses were performed by one-way ANOVA followed by Bonferroni multiple comparison* post hoc* test. SC = sham control; tPA = tissue plasminogen activator. *n* = 6 for each group.

By day 14 after CLI induction, the numbers of cells positive for CXCR4 and SDF-1*α* in groups 1 and 4 were highest in their CLI + tPA subgroups and lowest in the SC subgroups and significantly higher in the CLI subgroups than those in their SC + tPA subgroups (Figures 5 and 6). Moreover, the number of cells positive for these two markers in group 2 was significantly higher in the CLI and CLI + tPA subgroups than that in the SC and SC + tPA subgroups, but there was no notable difference between the former two or the latter two subgroups (Figures 5 and 6). On the other hand, no significant difference was noted in the number of cells positive for the two markers among the four subgroups of group 3.

**Figure 7:**
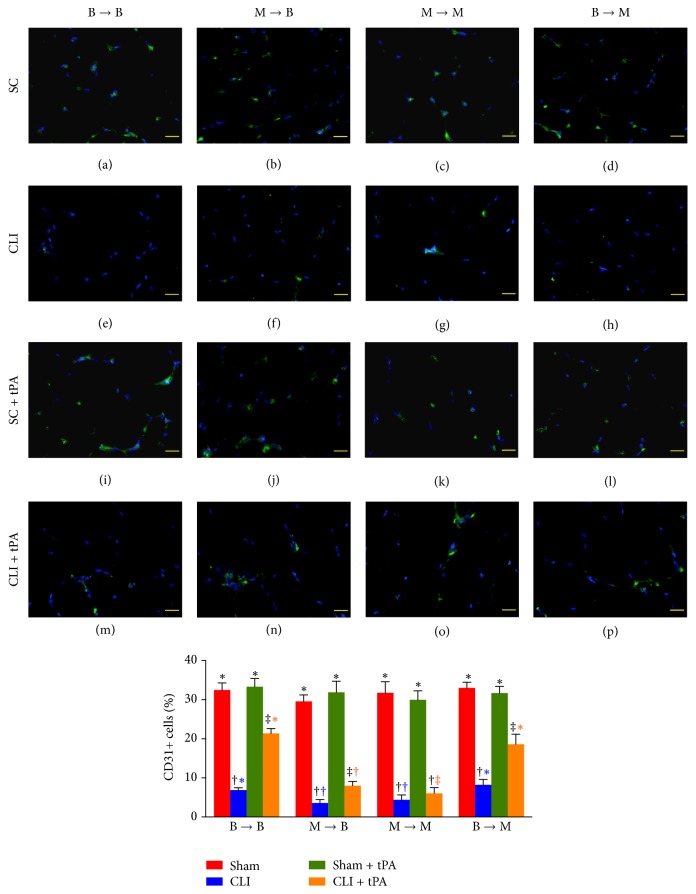
Immunofluorescent (IF) microscopic findings (400x) of CD31+ cells in ischemic quadriceps at day 14 after critical limb ischemia (CLI) induction. (a–p) IF microscopic findings of CD31+ cells in ischemic area in four treatment conditions of four groups. (1) Statistical analysis for subgroups of each group. (1) In B → B group, subgroups with different black symbols (*∗*, †, and ‡), *p* < 0.05. (2) In M → B group, subgroups with different black symbols (*∗*, †, and ‡), *p* < 0.05. (3) In M → M group, subgroups with different black symbols (*∗*, †), *p* < 0.05. (4) In B → M group, subgroups with different black symbols (*∗*, †, and ‡), *p* < 0.05. (2) Statistical analysis for group comparison with respective treatment condition. (1) In SC subgroup (red bar chart), *p* > 0.1. (2) In CLI subgroup (blue bar chart), groups with different blue symbols (*∗*, †), *p* < 0.05. (3) In SC + tPA subgroup (green bar chart), *p* > 0.1. (4) In CLI + tPA subgroup (orange bar chart), groups with different orange symbols (*∗*, †, and ‡), *p* < 0.05. All statistical analyses were performed by one-way ANOVA followed by Bonferroni multiple comparison* post hoc* test. SC = sham control; tPA = tissue plasminogen activator. *n* = 6 for each group.

**Figure 8:**
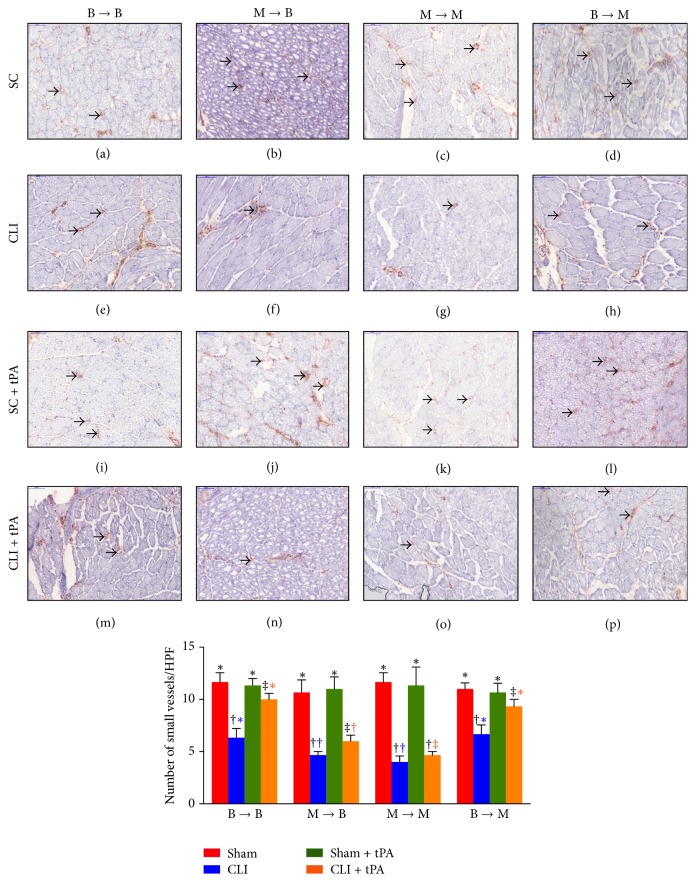
Immunohistochemical (IHC) staining for alpha-smooth muscle actin (*α*-SMA) to detect small vessels in ischemic quadriceps at day 14 after critical limb ischemia (CLI) induction. (a–p) IHC staining to examine the distribution of *α*-SMA + small vessels (black arrows) in ischemic area in four treatment conditions of four groups. (1) Statistical analysis for subgroups of each group. (1) In B → B group, subgroups with different black symbols (*∗*, †, and ‡), *p* < 0.05. (2) In M → B group, subgroups with different black symbols (*∗*, †, and ‡), *p* < 0.05. (3) In M → M group, subgroups with different black symbols (*∗*, †), *p* < 0.05. (4) In B → M group, subgroups with different black symbols (*∗*, †, and ‡), *p* < 0.05. (2) Statistical analysis for group comparison with respective treatment condition. (1) For SC subgroup (red bar chart), *p* > 0.05. (2) For CLI subgroup (blue bar chart), groups with different blue symbols (*∗*, †), *p* < 0.05. (3) For SC + tPA treatment condition (green bar chart), *p* > 0.05. (4) For CLI + tPA treatment condition (orange bar chart), groups with different orange symbols (*∗*, †, and ‡), *p* < 0.05. All statistical analyses were performed by one-way ANOVA followed by Bonferroni multiple comparison* post hoc* test. SC = sham control; tPA = tissue plasminogen activator. *n* = 6 for each group.

Furthermore, the numbers of CD31+ (an indicator of endothelial cells) and *α*-SMA+ (an indicator of small vessels) cells in groups 1, 2, and 4 were significantly higher in the SC and SC + tPA subgroups than those in the CLI and CLI + tPA subgroups and significantly higher in the CLI + tPA than in the CLI only subgroups, but no significant difference was noted between the SC and SC + tPA subgroups (Figures 7 and 8). In group 3, the number of cells positive for the two biomarkers was higher in the SC and SC + tPA subgroups than that in the CLI and CLI + tPA subgroups, but there was no notable difference between the former two and the latter two subgroups (Figures 7 and 8).

### 3.7. Immunostaining for Identifications of Angiogenesis Cells and Small Vessels in Ischemic Quadriceps on Day 14 after CLI Induction: Subgroup Analysis

By day 14 after CLI induction, in the SC subgroups, the number of CXCR4+ cells (Figure 5), SDF-1*α*+ cells (Figure 6), CD31+ cells (Figure 7), and *α*-SMA+ small vessels (Figure 8) did not differ among the four groups. On the other hand, for the CLI only, SC + tPA, and CLI + tPA subgroups, the number of CXCR4+, SDF-1*α*+, CD31+, and *α*-SMA+ small vessels was significantly higher in groups 1 and 4 than that in groups 2 and 3, but no difference was noted among the respective subgroups between groups 1 and 4. However, the number of cells positive for the four biomarkers was significantly higher in group 2 than that in group 3 only in the CLI + tPA subgroup.

### 3.8. Protein Expressions of Proangiogenic Factors in Ischemic Quadriceps on Day 14 after CLI Procedure: Subgroup Analysis

**Figure 9:**
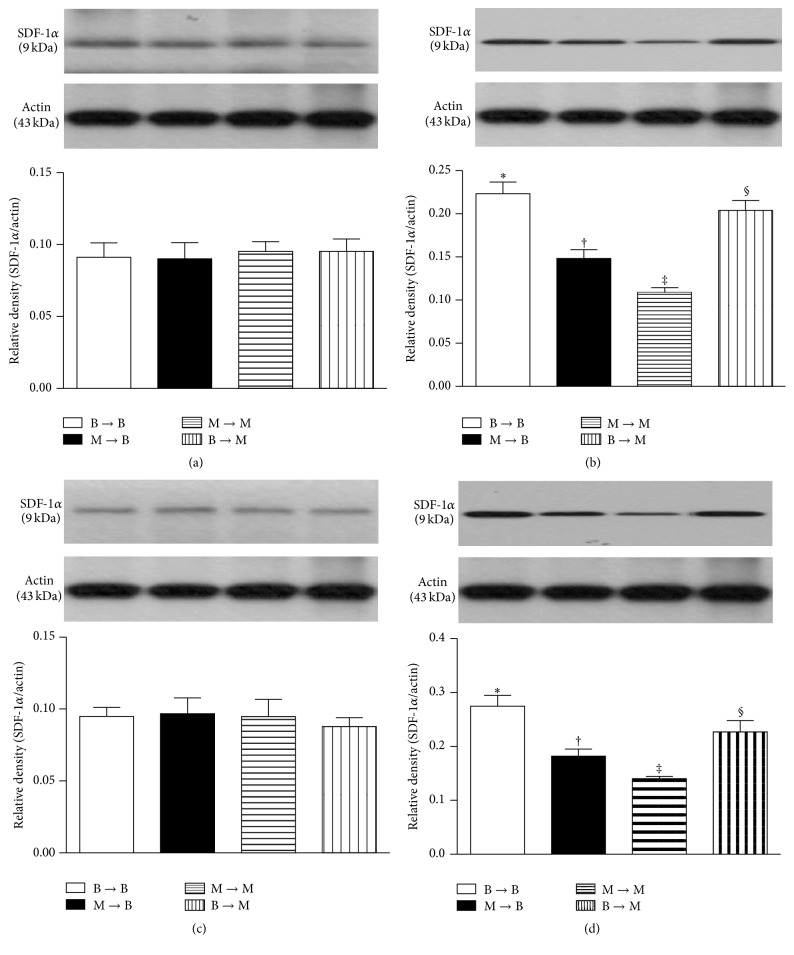
Protein expression of stromal cell-derived factor- (SDF-) 1*α* in ischemic quadriceps at day 14 after critical limb ischemia (CLI) induction. (a) At sham control (SC) treatment condition, SDF-1*α* did not differ among four groups, *p* = 1.0. (b) At CLI subgroup, groups with different symbols (*∗*, †, ‡, and §), *p* < 0.05. (c) At SC + tPA treatment condition, SDF-1*α* did not differ among four groups, *p* = 1.0. (d) At CLI + tPA subgroup, groups with different symbols (*∗*, †, ‡, and §), *p* < 0.05. All statistical analyses were performed by one-way ANOVA followed by Bonferroni multiple comparison* post hoc* test. tPA = tissue plasminogen activator. *n* = 6 for each group.

**Figure 10:**
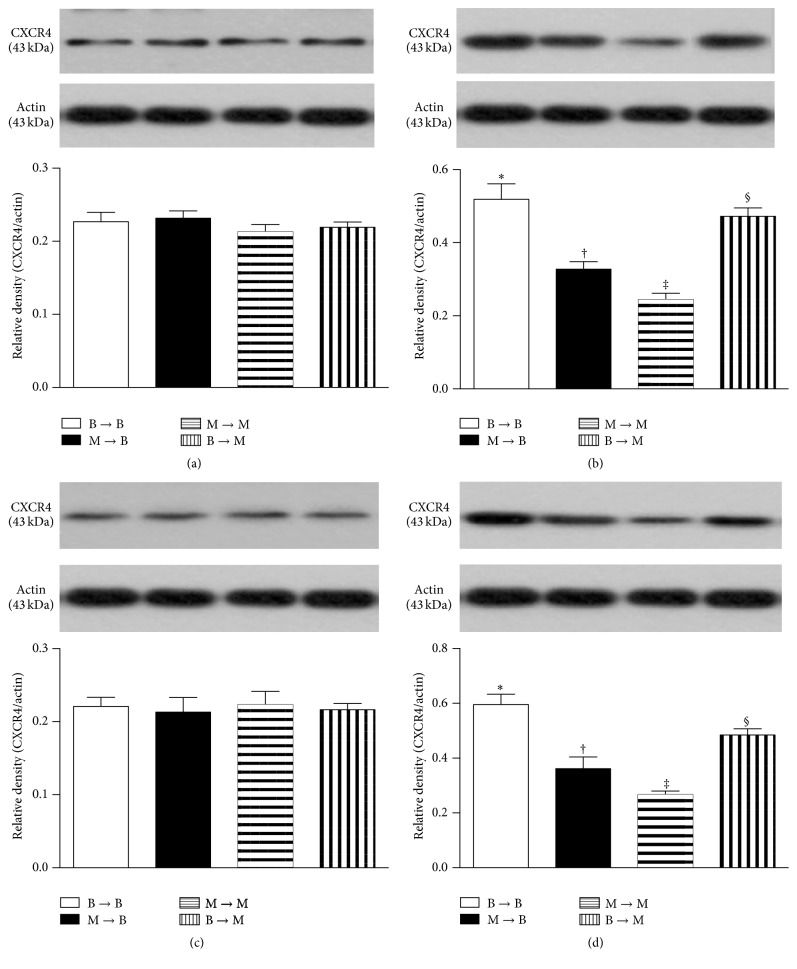
Protein expression of CXCR4 in ischemic quadriceps at day 14 after critical limb ischemia (CLI) induction. (a) At sham control (SC) treatment condition, CXCR4 did not differ among four groups, *p* = 1.0. (b) At CLI subgroup, groups with different symbols (*∗*, †, ‡, and §), *p* < 0.05. (c) At SC + tPA subgroup, CXCR4 did not differ among four groups, *p* = 1.0. (d) At CLI + tPA subgroup, groups with different symbols (*∗*, †, ‡, and §), *p* < 0.05. All statistical analyses were performed by one-way ANOVA followed by Bonferroni multiple comparison* post hoc* test. tPA = tissue plasminogen activator. *n* = 6 for each group.

**Figure 11:**
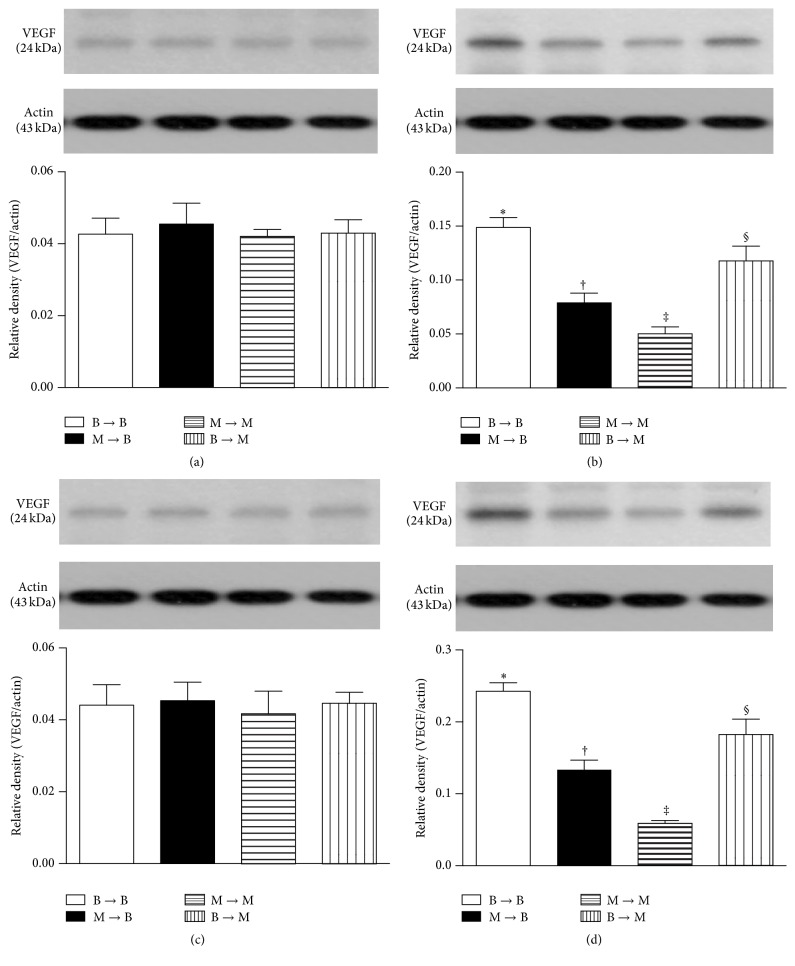
Protein expression of vascular endothelial growth factor (VEGF) in ischemic quadriceps at day 14 after critical limb ischemia (CLI) induction. (a) At sham control (SC) treatment condition, VEGF did not differ among four groups, *p* = 1.0. (b) At CLI subgroup, groups with different symbols (*∗*, †, ‡, and §), *p* < 0.05. (c) At SC + tPA subgroup, VEGF did not differ among four groups, *p* = 1.0. (d) At CLI + tPA subgroup, groups with different symbols (*∗*, †, ‡, and §), *p* < 0.05. All statistical analyses were performed by one-way ANOVA followed by Bonferroni multiple comparison* post hoc* test. tPA = tissue plasminogen activator. *n* = 6 for each group.

**Figure 12:**
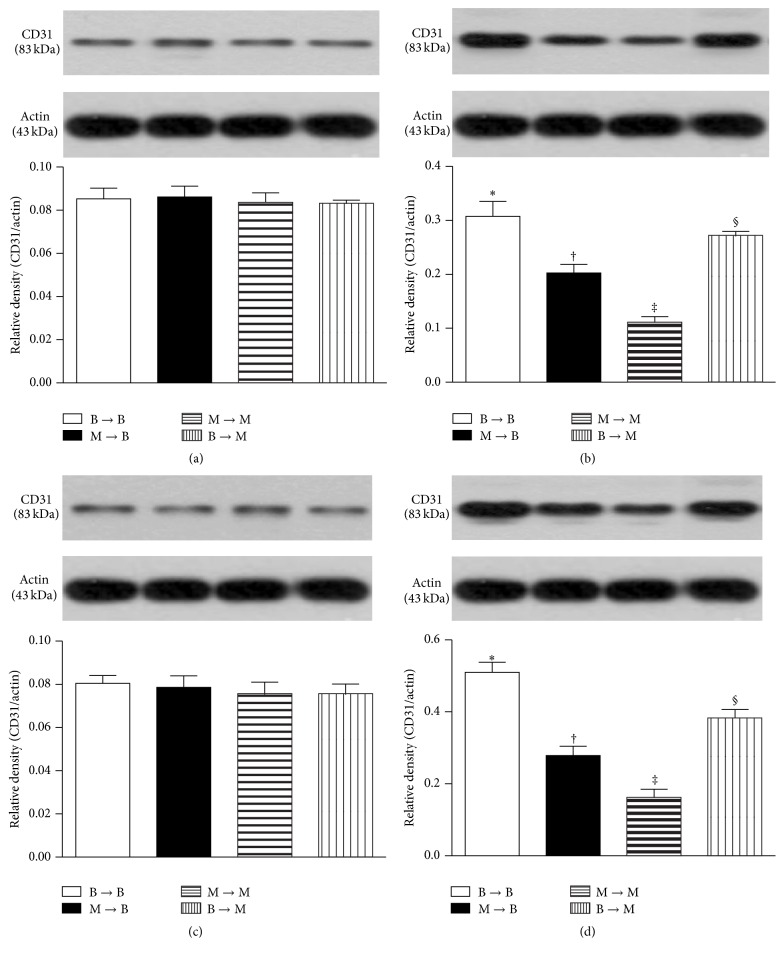
Protein expression of CD31 in ischemic quadriceps at day 14 after critical limb ischemia (CLI) induction (*n* = 6). (a) At sham control (SC) treatment condition, CD31 did not differ among four groups, *p* = 1.0. (b) At CLI subgroup, groups with different symbols (*∗*, †, ‡, and §), *p* < 0.05. (c) At SC + tPA treatment condition, CD31 did not differ among four groups, *p* = 1.0. (d) At CLI + tPA subgroup, groups with different symbols (*∗*, †, ‡, and §), *p* < 0.05. All statistical analyses were performed by one-way ANOVA followed by Bonferroni multiple comparison* post hoc* test. tPA = tissue plasminogen activator. *n* = 6 for each group.

In the SC and SC + tPA subgroups, the protein expressions of SDF-1*α* (Figure 9), CXCR4 (Figure 10), VEGF (Figure 11), and CD31 (Figure 12), four indicators of angiogenesis, were similar among the four groups. Additionally, the protein expressions of these parameters did not differ between the two subgroups.

In the CLI and CLI + tPA subgroups, the protein expressions of these four biomarkers were highest in group 1 and lowest in group 3 and significantly higher in group 4 than those in group 2.

### 3.9. Immunofluorescent Staining for Identifications of Neutrophils and Macrophages in Ischemic Quadriceps on Day 14 after CLI Induction: Group Analysis

**Figure 13:**
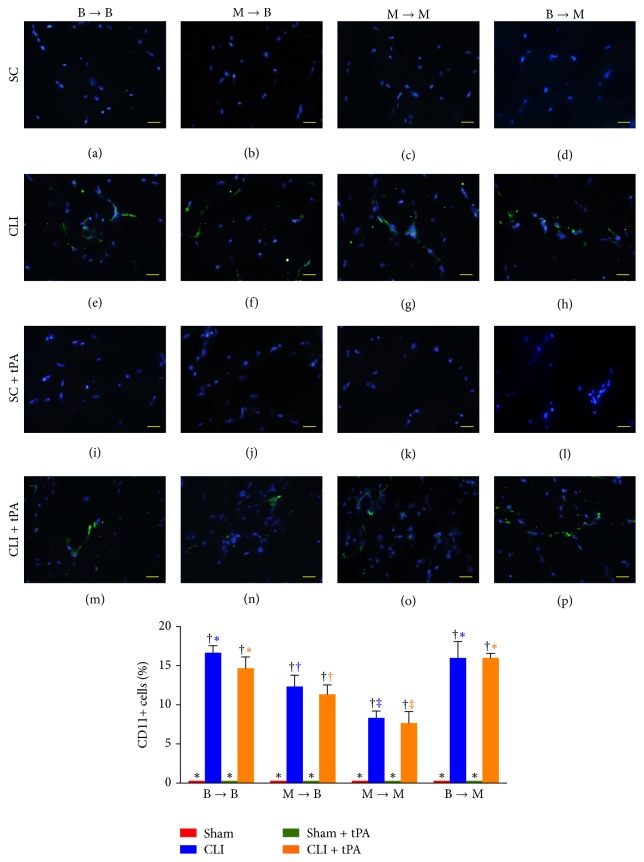
Immunofluorescent (IF) microscopic findings (400x) of neutrophils (CD11+ cells) in ischemic quadriceps at day 14 after critical limb ischemia (CLI) induction. (a–p) IF microscopic findings of neutrophils in ischemic area in four treatment conditions of four groups. (1) Statistical analysis for subgroups of each group. (1) In B → B group, subgroups with different black symbols (*∗*, †), *p* < 0.05. (2) In M → B group, subgroups with different black symbols (*∗*, †), *p* < 0.05. (3) In M → M group, subgroups with different black symbols (*∗*, †), *p* < 0.05. (4) In B → M group, subgroups with different black symbols (*∗*, †), *p* < 0.05. (2) Statistical analysis for group comparison with respective treatment condition. (1) In SC subgroup (red bar chart), *p* > 0.05. (2) In CLI subgroup (blue bar chart), groups with different blue symbols (*∗*, †, and ‡), *p* < 0.05. (3) In SC + tPA subgroup (green bar chart), *p* > 0.05. (4) In CLI + tPA subgroup (orange bar chart), groups with different orange symbols (*∗*, †, and ‡), *p* < 0.05. All statistical analyses were performed by one-way ANOVA followed by Bonferroni multiple comparison* post hoc* test. SC = sham control; tPA = tissue plasminogen activator. *n* = 6 for each group.

**Figure 14:**
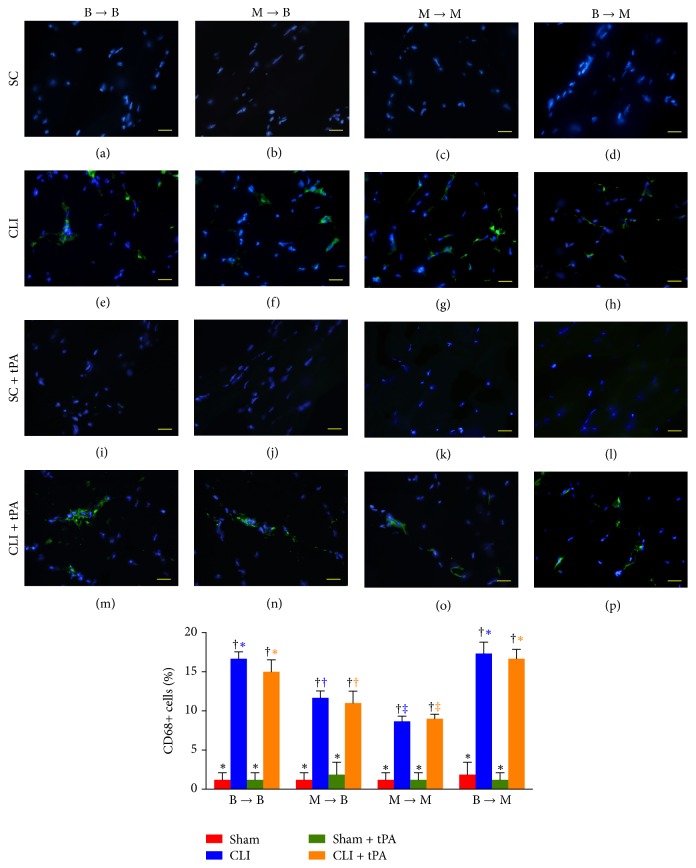
Immunofluorescent (IF) microscopic findings (400x) of macrophages (CD68+ cells) in ischemic quadriceps at day 14 after critical limb ischemia (CLI) induction. (a–p) IF microscopic findings of CD68+ cells in ischemic area in four treatment conditions of four groups. (1) Statistical analysis for subgroups of each group. (1) In B → B group, subgroups with different black symbols (*∗*, †), *p* < 0.05. (2) In M → B group, subgroups with different black symbols (*∗*, †), *p* < 0.05. (3) In M → M group, subgroups with different black symbols (*∗*, †), *p* < 0.05. (4) In B → M group, subgroups with different black symbols (*∗*, †), *p* < 0.05. (2) Statistical analysis for group comparison with respective treatment condition. (1) In SC subgroup (red bar chart), *p* > 0.05. (2) In CLI subgroup (blue bar chart), groups with different blue symbols (*∗*, †, and ‡), *p* < 0.05. (3) In SC + tPA subgroup (green bar chart), *p* > 0.05. (4) In CLI + tPA subgroup (orange bar chart), groups with different orange symbols (*∗*, †, and ‡), *p* < 0.05. All statistical analyses were performed by one-way ANOVA followed by Bonferroni multiple comparison* post hoc* test. SC = sham control; tPA = tissue plasminogen activator. *n* = 6 for each group.

By day 14 after CLI induction, the numbers of infiltrated neutrophils (CD11+ cells) and macrophages (CD68+ cells) in ischemic quadriceps in all groups were higher in CLI and CLI + tPA subgroups than those in SC and SC + tPA subgroups (Figures 13 and 14). Moreover, in all groups, the number of cells positive for these two markers showed no significant difference between CLI and CLI + tPA subgroups (Figures 13 and 14). In addition, no difference was noted in the number of cells positive for the two markers between SC and SC + tPA subgroups of all groups (Figures 13 and 14).

### 3.10. Immunofluorescent Staining for Identifications of Neutrophils and Macrophages in Ischemic Quadriceps on Day 14 after CLI Induction: Subgroup Analysis

By day 14 after CLI induction, in the SC and SC + tPA subgroups, the number of neutrophils (CD11+ cells) and macrophages (CD68+ cells) did not differ among the four groups. On the other hand, for the CLI and CLI + tPA subgroups, the number of neutrophils and macrophages was significantly higher in groups 1 and 4 than that in groups 2 and 3, but no difference was noted among the respective subgroups between groups 1 and 4. However, in the CLI and CLI + tPA subgroups, the number of macrophages and neutrophils was higher in group 2 than that in group 3.

### 3.11. MMP-9 Activity in Bone Marrow

**Figure 15:**
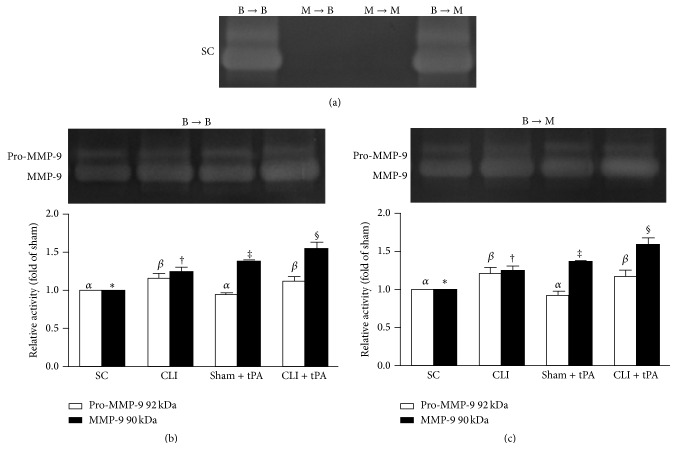
Matrix metalloproteinase- (MMP-) 9 activity in BM by 18 h after CLI procedure. (a) Zymography analysis of matrix metalloproteinase- (MMP-) 9 activity in BM of four groups. No MMP-9 activity was identified in M → M and M → B groups. On the other hand, MMP-9 activity was observed in B → B and B → M groups. (b) (1) Zymography analysis of pro-MMP-9 activity in B → B group of different treatment condition (white bar chart), *α* versus *β*, *p* < 0.05. (2) Zymography analysis of active form of MMP-9 in B → B group of different treatment condition (black bar chart), subgroups with different symbols (*∗*, †, ‡, and §), *p* < 0.05. Symbols (*∗*, †, ‡, and §) indicate significance (at 0.05 level). *n* = 4 for each group. (c) (1) Zymography analysis of pro-MMP-9 activity in B → M group of different treatment condition (white bar chart), *α* versus *β*, *p* < 0.05. (2) Zymography analysis of active form of MMP-9 in B → M group of different treatment condition (black bar chart), subgroups with different symbols (*∗*, †, ‡, and §), *p* < 0.05. *n* = 4 for each group.

As expected, zymography analysis revealed no MMP-9 activity in BM in groups 2 and 3 (Figure 15(a)). On the other hand, as compared with groups 2 and 3, MMP-9 activity in BM was remarkably increased in groups 1 and 4 (Figure 15(a)). Additionally, the expression of this activated form of MMP-9 was highest in the CLI + tPA and lowest in the SC subgroups and significantly higher in the SC + tPA than in the CLI subgroups for groups 1 (Figure 15(b)) and 4 animals (Figure 15(c)).

## 4. Discussion

The aim of this study, which is a continuation of two of our recent reports [7, 8], was to investigate the role of tPA-MMP-9 axis in angiogenesis and enhancement of blood flow through mobilization of EPCs from BM to circulation and CLI region. There are several notable implications from the results of the present study. First, using the BM transplantation technique with MMP-9 deficient mice, we proved a crucial role of MMP-9 in EPC mobilization from BM to circulation and homing to CLI region for enhancement of angiogenesis and restoration of blood flow. Second, tPA plays a unique role in increasing the numbers of EPCs in BM environment and circulation through enhancing MMP-9 activity in BM. Third, bolus exogenous tPA appears to be effective for further upregulating MMP-9 activity which in turn increases BM and circulating levels of EPCs. Finally, the tPA-MMP-9 signaling axis is like a lock (i.e., MMP-9) and key (i.e., tPA) that cooperate and coordinate the numbers of EPCs in BM and circulation.

By day 2 after CLI induction, the ratio of INBF was similar among all groups of animals regardless of their treatment subgroups. These findings are consistent with those of our recent studies showing that a boost of tPA 3 h after CLI induction did not significantly restore the blood flow in ischemic region through thrombolysis [7, 8]. However, when we look at the results of day 14 after CLI induction in the CLI only and CLI + tPA subgroups, the ratio of INBF was significantly higher in WT mice receiving BM transplantation from WT donors (group 1) (i.e., WT to WT) and MMP-9^−/−^ mice receiving BM transplantation from WT donors (group 4) (WT to MMP-9^−/−^) than that in WT mice (i.e., wild-type mice) receiving BM transplantation from MMP-9^−/−^ donors (group 2) (i.e., MMP-9^−/−^ to WT) and MMP-9^−/−^ mice receiving BM transplantation from MMP-9^−/−^ donors (group 3) (i.e., MMP-9^−/−^ to MMP-9^−/−^) (Figure 4). In addition, similar to our previous finding [7], the blood flow recovery in the CLI subgroups was not as conspicuous as the CLI + tPA subgroup. Although several studies have indicated that MMP-9 plays an essential role in modulating the homing and angiogenic activity of bone marrow-derived EPCs under ischemic stress [5, 14, 18], the upstream trigger of MMP-9 is not fully identified. In our recent studies, we have shown that tPA therapy can significantly improve blood flow in a mouse CLI model only in the presence of MMP-9 [7, 8]. This result suggested that tPA might be an upstream trigger of MMP-9 signaling. Of importance, MMP-9 is expressed in several types of cells involved in limb ischemia and followed recovery, including muscle cells, fibroblasts, endothelial cells, and bone marrow cells [19, 20]. Besides, the role of MMP-9 postischemic injury remains controversial. The use of MMP inhibitors to improve outcome in ischemic stroke and acute myocardial infarction AMI has been investigated [21, 22], while neutrophil-derived MMP-9 was also indicated to trigger aortic dissection [23]. Another study also demonstrated that loss of MMP-9 impaired the blood flow recovery after CLI [5, 24]. Hence, the roles MMP-9 plays among different tissues might be distinct. In this study, we found that the transplantation of wild-type bone marrow cells rescued the impaired blood flood recovery in MMP-9^−/−^ mice after CLI (Figure 4). This finding indicated that the bone marrow-specific expression of MMP-9 is not only critical for migration of EPCs, but also important in angiogenesis and blood flow recovery after CLI. Our findings echoed a previous study regarding the effects of MMP-9 released from bone marrow-derived cells on the progression of BBB disruption in the ischemic brain [25].

A previous finding also demonstrated that the wild-type bone marrow transplantation could rescue the impairment of blood flow recovery in MMP-9 deficient mice with CLI, even as equivalent to that in wild-type mice [24]. Therefore, despite the role of MMP-9 in noncirculating cells, MMP-9 expression in bone marrow-derived cells, such as myeloid, lymphoid, or endothelial lineage cells, is crucial for angiogenesis and blood flow recovery after CLI. Results from immunofluorescent staining also indicated that the infiltration of macrophages and neutrophils in ischemic quadriceps is reduced by loss of MMP-9 (Figures 13 and 14). Hence, it is reasonable that although MMP-9 deficiency impairs endothelial sprouting ability in aortic ring assay (Figure 1), wild-type bone marrow transplantation could revert the reduced blood flow recovery in CLI MMP-9^−/−^ mice* in vivo *(Figure 4).

To elucidate whether the functional outcomes of CLI were correlated to the circulating and BM levels of EPCs, EPCs were quantified by flow cytometry through identification of their surface markers. One principal finding in the present study is that, in both normal and CLI conditions, circulating numbers of EPCs (i.e., c-Kit+/CD31+ and Sca-1+/KDR+ cells) were remarkably lower in WT or MMP-9^−/−^ mice receiving BM transplantation from MMP-9^−/−^ donors (groups 2 and 3) than those receiving BM transplantation from WT donors (groups 1 and 4) (Figure 2). However, the alternation of EPC population in bone marrow showed an opposite trend (Figure 3). Interestingly, these biomarkers did not differ in BM or circulation in groups 1 and 4 animals (Figures 2 and 3). Additionally, zymographic study demonstrated that MMP-9 activity in BM was remarkably higher in groups 1 and 4 than that in groups 2 and 3 (Figure 15). Our findings, therefore, highlight that MMP-9 plays a pivotal role in regulating the kinetics of EPCs (i.e., mobilization from bone marrow to circulation).

Interestingly, our studies [7, 8] have shown that endogenous tPA was activated only under ischemic conditions in which plasminogen is cleaved to plasmin (i.e., the active form) that elicits MMP-9 activity in the BM, leading to the degradation of SDF-1*α* [7, 8]. This results in a concentration gradient of SDF-1*α* (i.e., lower in BM and higher in circulation) that leads to a mobilization of EPCs from BM to circulation [7, 8]. Besides, MMP-9 acts by cleaving the membrane-bound c-Kit-L to the soluble form c-Kit-L, which then interacts with the EPCs c-Kit receptor to initiate a signal (i.e., the downstream signaling of MMP-9) that is essential for BM-EPC differentiation and mobilization to systemic circulation [7–12]. Our present and recent findings [7, 8] as well as those from the other previous studies [9–12] all suggest an important role of the tPA-MMP-9 signaling axis in the coordination of EPC mobilization. However, MMP-9 can also be activated by other proteases, such as MMP-3 [26, 27]. The transcription level of MMP-9 is found to be upregulated by MMP-1 and MMP-3 in macrophages [28], while the activation of MMP-3 is associated with the activity of plasmin [26]. Hence, the interaction among plasmin and MMPs may have roles in tPA-MMP-9-mediated EPC mobilization and angiogenesis post-CLI.

To further confirm an upregulation of the expressions of proangiogenic factors in the CLI region in the presence of MMP-9 at protein and cellular levels, western blot and IF staining of the ischemic muscle were performed in the present study, respectively. A principal finding in the present study is that, for their SC and SC + tPA subgroups, the protein expressions of CXCR4, SDF-1*α*, and CD31 in CLI region did not differ among the four groups of animals. These findings suggest that, in the absence of ischemic stimulus, BM transplantation did not contribute to the enhancement of protein expressions of proangiogenic factors in remote region. Of importance is that the protein expressions of these factors in the CLI and CLI + tPA subgroups were significantly higher in groups 1 and 4 than those in groups 2 and 3. Besides, IF microscopic findings showed that, in the CLI and CLI + tPA subgroups, the pattern of cellular expressions of proangiogenic biomarkers (CXCR4+, SDF-1*α*+, CD31+, and *α*-SMA+ small vessels) was identical to that of their protein expressions in the four groups of animals. These findings, in addition to strengthening those of our recent studies demonstrating that tPA treatment augmented the protein and cellular expressions of proangiogenic biomarkers in the setting of CLI [7, 8], may also explain the remarkable improvement in blood flow in the ischemic zone in groups 1 and 4 by day 14 after CLI induction. Besides, the results of the present study provide two novel findings: (1) tPA upregulated the expressions of proangiogenic factors only in the presence of MMP-9 under ischemic condition; (2) tPA therapy may be beneficial for patients with acute myocardial infarction and acute ischemic stroke other than its role in enhancing reperfusion through the mechanism of thrombolysis.

This study has limitations. First, this study did not directly confirm the hypothesis that circulating EPCs were mobilized solely from the BM. The distribution of EPC was not directly observed in the ischemic region of mice with CLI to clarify the correlation between circulation EPC and tissue EPC. Besides, we did not perform double knockout experiments (i.e., knockout of tPA and MMP-9 in the same animal) to further establish the role of the tPA-MMP-9 signaling axis in controlling EPC kinesis.

## 5. Conclusion

**Figure 16:**
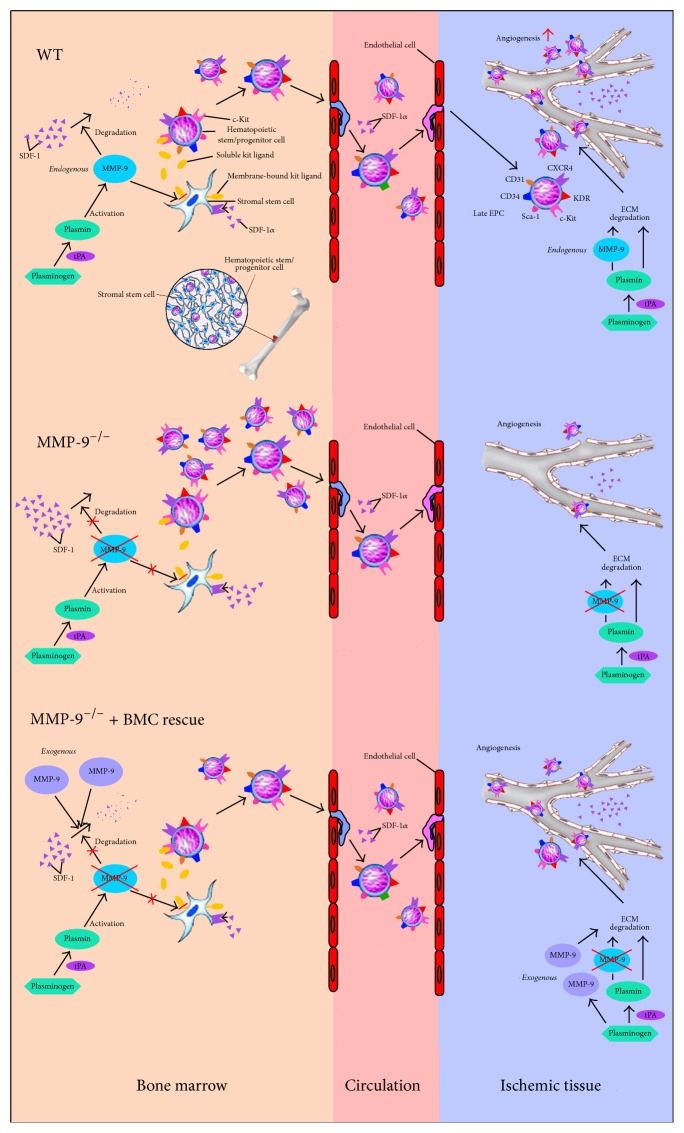
Proposed mechanisms. The proposed rescue model of bone marrow cells (BMC) transplantation on enhancing angiogenesis in matrix metalloproteinase- (MMP-) 9 deficient mice with critical limb ischemia. EPC = endothelial progenitor cells; SDF-1*α* = stromal cell-derived factor-1*α*; ECM = extracellular matrix.

MMP-9 plays a unique role in EPC mobilization from BM to circulation and homing to ischemic zone. Moreover, tPA is essential for eliciting MMP-9 activity. The proposed mechanisms have been summarized in Figure 16. The findings of the present study, therefore, provide important evidence for understanding the role of the tPA-MMP-9 signaling axis in the regulation of the kinetics of EPCs in a murine experimental setting of CLI.

## Supplementary Material

Supplemental Figure 1: Experimental grouping and design of present study. Upper panel: The schematic illustration of the grouping for the bone marrow cell (BMC) transplantation model. Lower panel: The schematic illustration of experimental grouping and procedures for BMC transplantation, critical limb ischemia (CLI) induction, blood flow observation, and sample collection. MMP-9, matrix metalloproteinase-9; PB, peripheral blood; BM, bone marrow.
