# Supplementary material for: tPA-MMP-9 Axis Plays a Pivotal Role in Mobilization of Endothelial Progenitor Cells from Bone Marrow to Circulation and Ischemic Region for Angiogenesis
Source: Stem Cells Int. 2016 Aug 16;2016:5417565. doi: 10.1155/2016/5417565 (PMC5004042; doi:10.1155/2016/5417565)
Supplement: Supplementary file 1 — Supplemental Figure 1: Experimental grouping and design of present study. Upper panel: The schematic illustration of the grouping for the bone marrow cell (BMC) transplantation model. Lower panel: The schematic illustration of experimental grouping and procedures for BMC transplantation, critical limb ischemia (CLI) induction, blood flow observation, and sample collection. MMP-9, matrix metalloproteinase-9; PB, peripheral blood; BM, bone marrow. [file 5417565.f1.pdf]

Supplemental data

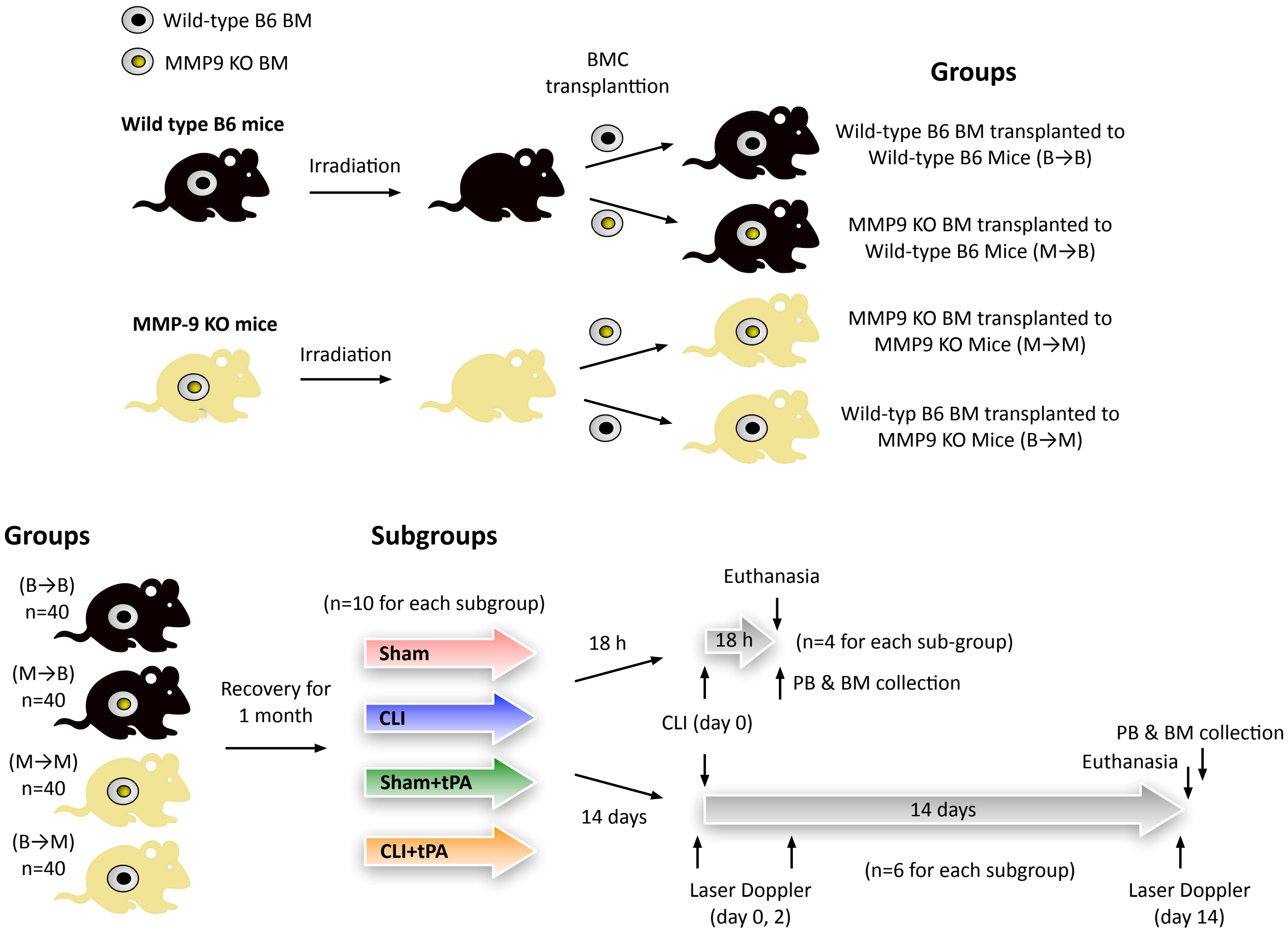

Supplemental Figure 1

Experimental grouping and design of present study. Upper panel: The schematic illustration of the grouping for the bone marrow cell (BMC) transplantation model. Lower panel: The schematic illustration of experimental grouping and procedures for BMC transplantation, critical limb ischemia (CLI) induction, blood flow observation, and sample collection. MMP-9, matrix metalloproteinase-9; PB, peripheral blood; BM, bone marrow.
